# Murine Type III interferons are functionally redundant and correlate with bacterial burden during influenza/bacterial super-infection

**DOI:** 10.1371/journal.pone.0255309

**Published:** 2021-10-07

**Authors:** Helen E. Rich, Danielle Antos, Collin C. McCourt, Wen Quan Zheng, Louis J. Devito, Kevin J. McHugh, Radha Gopal, Jieru Wang, John F. Alcorn

**Affiliations:** 1 Department of Immunology, University of Pittsburgh School of Medicine, Pittsburgh, PA, United States of America; 2 Department of Pediatrics, Children’s Hospital of Pittsburgh of UPMC, Pittsburgh, PA, United States of America; University of Alabama at Birmingham, UNITED STATES

## Abstract

**Background:**

Type III interferon, or interferon lambda (IFNλ) is a crucial antiviral cytokine induced by influenza infection. While IFNλ is important for anti-viral host defense, published data demonstrate that IFNλ is pathogenic during influenza/bacterial super-infection. It is known that polymorphisms in specific IFNλ genes affect influenza responses, but the effect of IFNλ subtypes on bacterial super-infection is unknown.

**Methods:**

Using an established model of influenza, *Staphylococcus aureus* super-infection, we studied IFNλ3^-/-^ and control mice to model a physiologically relevant reduction in IFNλ and to address its role in super-infection.

**Results:**

Surprisingly, IFNλ3^-/-^ mice did not have significantly lower total IFNλ than co-housed controls, and displayed no change in viral or bacterial clearance. Importantly, both control and IFNλ3^-/-^ mice displayed a positive correlation between viral burden and total IFNλ in the bronchoalveolar lavage during influenza/bacterial super-infection, suggesting that higher influenza viral burden drives a similar total IFNλ response regardless of IFNλ3 gene integrity. Interestingly, total IFNλ levels positively correlated with bacterial burden, while viral burden and bronchoalveolar lavage cellularity did not.

**Conclusions:**

These data suggest IFNλ2 can compensate for IFNλ3 to mount an effective antiviral and defense, revealing a functional redundancy in these highly similar IFNλ subtypes. Further, the IFNλ response to influenza, as opposed to changes in cellular inflammation or viral load, significantly correlates with susceptibility to bacterial super-infection. Moreover, the IFNλ response is regulated and involves redundant subtypes, suggesting it is of high importance to pulmonary pathogen defense.

## Introduction

Type III interferon (interferon lambda, IFNλ) is a family of anti-viral cytokines, composed of four subtypes in humans (IFNλ1–4, alternatively IL-29 and IL-28A/B). IFNλ has been shown to be highly important in pulmonary defense against influenza infection [1], and is the predominant interferon induced during infection the lung [2]. Seasonal influenza infection is responsible for 30–60,000 deaths in the United States each year, and killed an estimated 50 million people worldwide during the 1918 “Spanish Flu” pandemic [3]. A significant cause of influenza-related mortality is bacterial super-infection. Bacteria were isolated from 95% of tissue specimens from the 1918 pandemic [4], and bacterial super-infection continues to be present in almost half of pediatric deaths from influenza today [5]. While *Streptococcus pneumoniae* has classically been thought to be the most important super-infecting pathogen during influenza infection [6], data from influenza-associated pediatric deaths in recent years demonstrate a switch to *Staphylococcus aureus* as the predominant super-infecting pathogen [5]. Importantly, this phenomenon of *S*. *aureus* super-infection during influenza has been robustly modeled in mice. When mice are infected with influenza five to seven days preceding *S*. *aureus* challenge, they have significantly higher levels of bacteria in the lung and increased mortality compared with mice infected only with *S*. *aureus* [7].

While IFNλ is important in pulmonary defense against influenza, data from mice lacking the IFNλ receptor show that IFNλ signaling ablation reduces bacterial burden and improves survival during influenza super-infection with methicillin-resistant *S*. *aureus* (MRSA) or *S*. *pneumoniae* [8,9]. Moreover, overexpression of IFNλ during influenza/MRSA super-infection results in increased MRSA burden [10]. Together, these studies suggest that IFNλ is an important regulator of susceptibility to bacterial super-infection during influenza. It is known that humans have polymorphic expression of the IFNλs, with IFNλ4 existing only as a pseudogene in many humans [11]. Polymorphisms in IFNλ3 (IL-28B) explain approximately half of the variation in response between people of European and African ancestries to pegylated-IFNα/ribavirin treatment for HCV [12]. While published data clearly demonstrate that IFNλ polymorphisms affect antiviral defense, there is no epidemiological data concerning the role of IFNλ polymorphisms in susceptibility to bacterial super-infection. Mice share two of the four human IFNλ family members, IFNλ2 (IL-28A) and IFNλ3 (IL-28B), and IFNλ3^-/-^ mice are commercially available. We hypothesized that IFNλ3^-/-^ mice would make a useful and physiologically relevant model for studying the effect of IFNλ subtypes on influenza/bacterial super-infection.

## Materials and methods

### Murine infections

**Fig 1:**
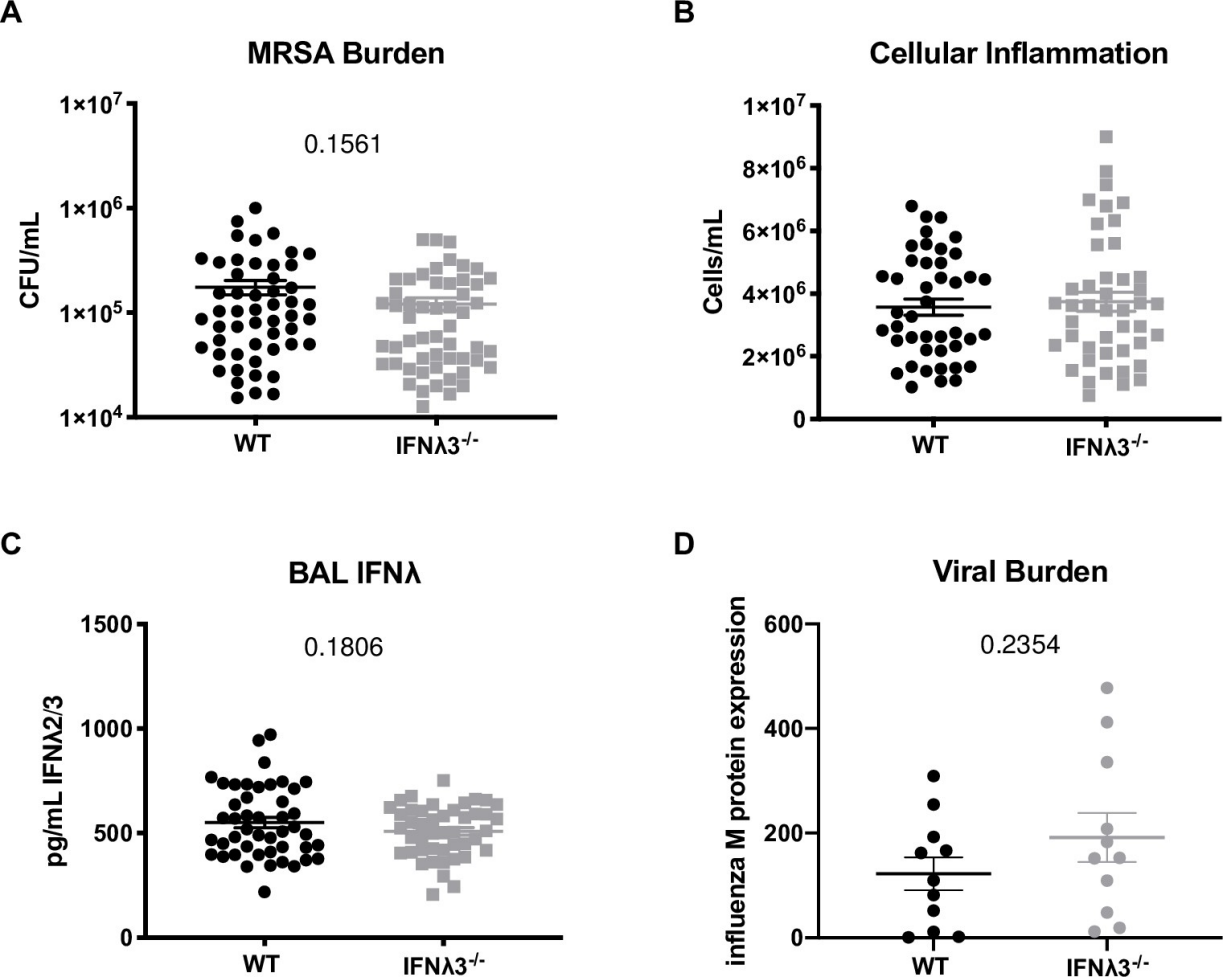
Absence of IFNλ3 does not alter influenza or bacterial clearance during super-infection. IFNλ3^-/-^ mice were bred in a homozygous manner in-house, and co-housed for one week with C57BL/6NJ controls (n = 3-5/group, nine independent experiments). On day 7 of co-housing, mice were infected with 25 PFU influenza A/PR/8/34 H1N1, six days later challenged with 5×10^7^ CFU USA300 MRSA, and harvested one day following bacterial challenge. **(A)** MRSA burden was measured by plating of lung homogenate and counting colony-forming units. **(B)** BAL cellularity was determined by counting total cells on a hemocytometer. **(C)** BAL IFNλ was quantified by ELISA for IFNλ2/3. **(D)** Viral burden was quantified by real-time qPCR for influenza M protein.

**Fig 2:**
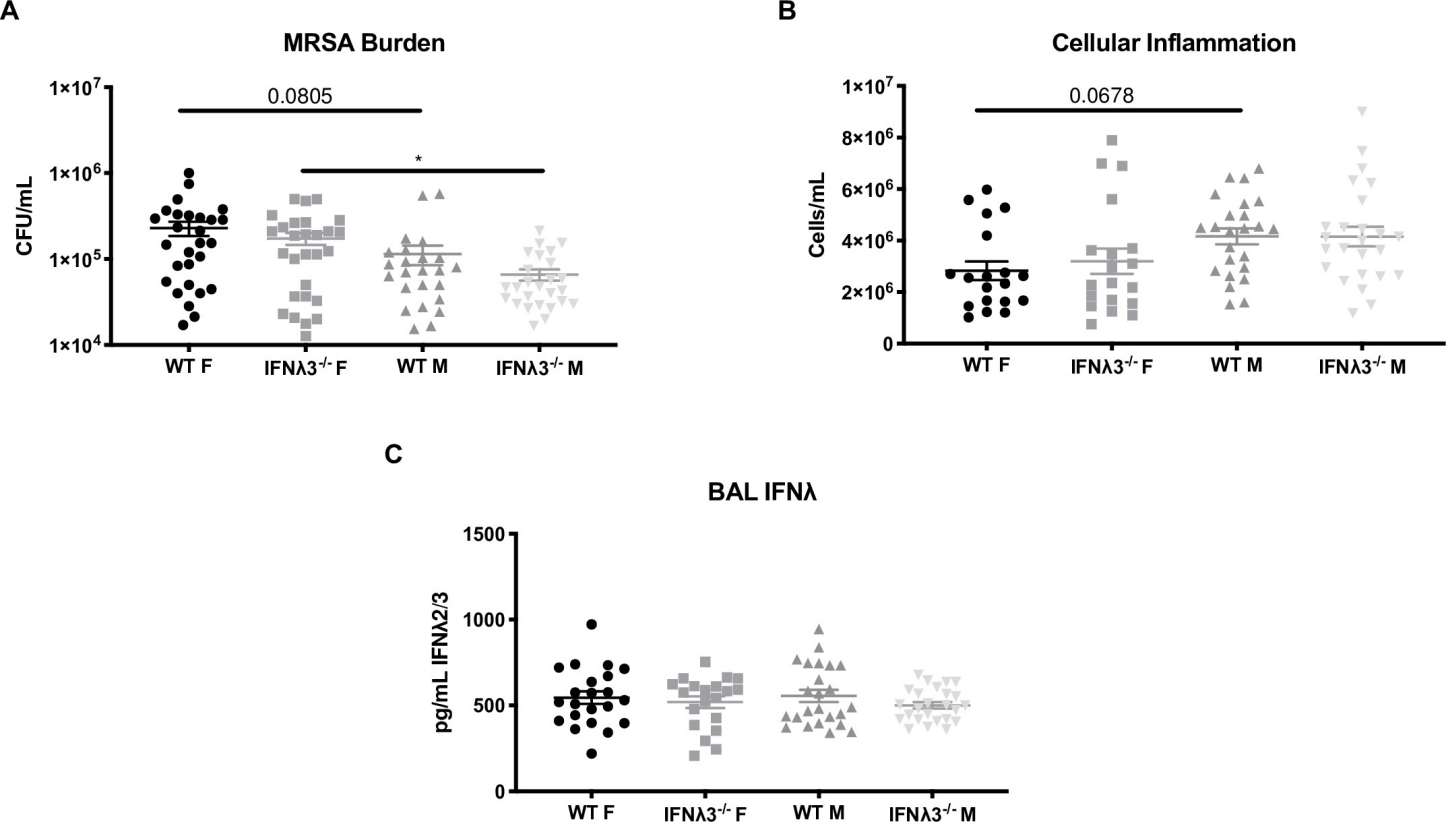
Female mice have higher bacterial burden than males during super-infection. IFNλ3^-/-^ mice and C57BL/6NJ controls were co-housed for one week, infected with 25 PFU influenza A/PR/8/34 H1N1, six days later challenged with 5×10^7^ CFU USA300 MRSA, and harvested one day following bacterial challenge. Data are the same as in Fig 1, but stratified by sex (n = 3-5/group, nine independent experiments). **(A)** MRSA burden was measured by plating of lung homogenate and counting colony-forming units. **(B)** BAL cellularity was determined by counting total cells on a hemocytometer. **(C)** BAL IFNλ was quantified by ELISA for IFNλ2/3.

**Fig 3:**
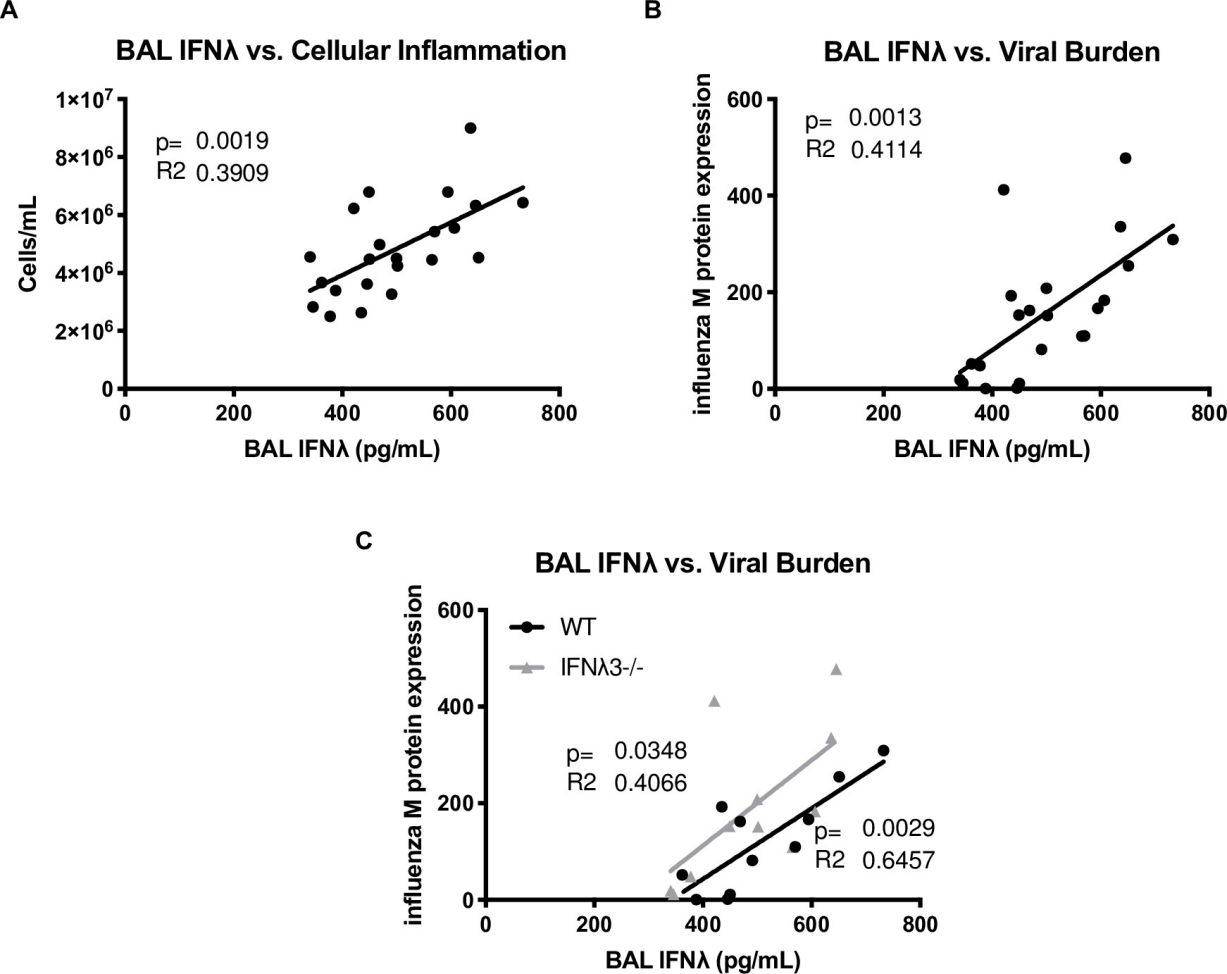
BAL IFNλ positively correlates with airspace inflammation and viral burden. IFNλ3^-/-^ mice and C57BL/6NJ controls were co-housed for one week, infected with 25 PFU influenza A/PR/8/34 H1N1, six days later challenged with 5×10^7^ CFU USA300 MRSA, and harvested one day following bacterial challenge (n = 2-5/group, six independent experiments). **(A)** BAL cellularity was determined by counting total cells on a hemocytometer. **(B-C)** Viral burden was quantified by real-time qPCR for influenza M protein.

**Fig 4:**
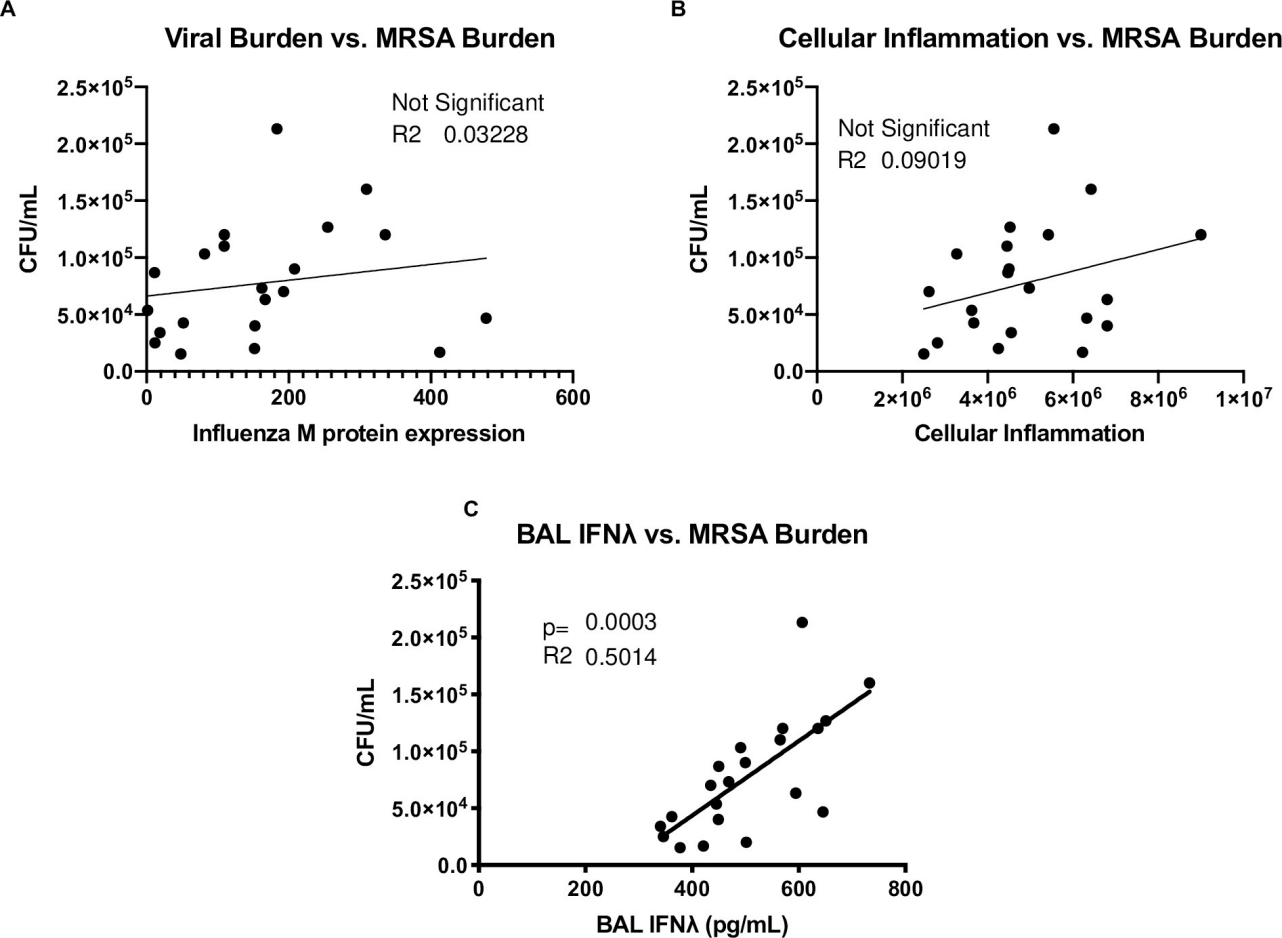
Airspace inflammation and viral burden do not independently correlate with bacterial burden. IFNλ3^-/-^ mice and C57BL/6NJ controls were co-housed for one week, infected with 25 PFU influenza A/PR/8/34 H1N1, six days later challenged with 5×10^7^ CFU USA300 MRSA, and harvested one day following bacterial challenge (n = 2-5/group, six independent experiments). MRSA burden was measured by plating of lung homogenate and counting colony-forming units. **(A)** Viral burden was quantified by real-time qPCR for influenza M protein. **(B)** BAL cellularity was determined by counting total cells on a hemocytometer. **(C)** BAL IFNλ was quantified by ELISA for IFNλ2/3.

IFNλ3^-/-^ mice on the C57BL/6NTac background (Ifnl3^tm1.1(KOMP)Vlcg^ mice) were a gracious gift of Dr. Jieru Wang and are currently cryoarchived in the NIH-supported Mutant Mouse Resource & Research Centers (MMRRC) as stock #048146-UCD. For Figs 1–3, IFNλ3^-/-^ mice were bred in-house in a homozygous manner and used for experiments at six to eight weeks of age. Six- to eight-week old C57BL/6NJ controls were ordered and co-housed with sex-matched IFNλ3^-/-^ mice for one week preceding influenza infection, for a total of two weeks of co-housing at harvest. For Fig 4, IFNλ3^-/-^ females were bred with C57BL/6NJ males, the resulting heterozygous F1 offspring were bred to each other, and knockout and wild-type (WT) mice from the F2 generation were used for experiments at six to eight weeks of age. Influenza A/PR/8/34 was originally provided by Dr. Kevan Hartshorn from Boston University and was propagated in MDCK cells as previously described [13]. This strain was originally isolated from humans in 1934 and was subsequently serially passaged in mice to increase virulence. No human subjects identifying information was available to the researchers. Mice were infected with 25 PFU influenza A/PR/8/34 H1N1, six days later challenged with 5×10^7^ colony forming unit (CFU) USA300 MRSA, and harvested one day later. All murine treatments (influenza and MRSA infections) were administered by oropharyngeal aspiration. Briefly, mice anesthetized with isoflurane were suspended by their top incisors from a string secured to a fixed board. The tongue was pulled out from the mouth using sterile blunt forceps, and 50 μL of inoculum was pipetted into the oropharynx. Mice were monitored for aspiration as evidenced by the disappearance of fluid at the back of the mouth, and a “clicking” noise as the fluid was aspirated into the lungs. All mice were maintained under pathogen-free conditions and all animal studies were conducted with approval from the University of Pittsburgh Institutional Animal Care and Use Committee. Mice were euthanized by pentobarbital injection followed by exsanguination by severing the renal artery. No mice died prior to planned euthanasia.

### Analysis of disease endpoints

At harvest, mouse lungs were lavaged with 1 ml sterile PBS without the addition of protease inhibitors. Bronchoalveolar lavage (BAL) fluid was centrifuged at 10,000×g for 5 min to pellet cells, and the supernatant was frozen for cytokine measurement by an enzyme-linked immunosorbent assay (ELISA) IFNλ3 (murine IL-28A/B DuoSet; R&D Systems, Minneapolis, MN). Total protein in BAL was quantified by the Pierce bicinchoninic acid (BCA) protein assay (ThermoFisher Scientific, Waltham, MA) Cell pellets from lavage fluid were resuspended in 500 μl sterile PBS and counted on a hemocytometer to enumerate infiltrating cells. The right upper lobe of each lung was mechanically homogenized and plated for bacterial CFU counting. Briefly, bacterial burden was determined by plating serial 10-fold dilutions of lung homogenate (right upper lobe of the lung homogenized in 1 ml sterile PBS). The right middle and lower lobes of each lung were snap-frozen in liquid nitrogen for RNA extraction suing the Absolutely RNA miniprep kit (Agilent Technologies, Santa Clara, CA). The left lobe of the lung was perfused with 10% formalin and sectioned for histological determination of inflammation. Perivascular, peribronchial, and parenchymal inflammation were determined by blinded scoring of hematoxylin and eosin-stained sections using a 1–4 scale. RNA was reverse transcribed into cDNA using the iScript cDNA synthesis kit (Bio-Rad, Hercules, CA), which was assayed by real-time PCR for gene expression with Assay on Demand TaqMan proprietary primer and probe sets (Life Technologies, Grand Island, NY). Viral burden was determined by RT-PCR for matrix protein using the following primers and probe—influenza M protein forward 5′-GGACTGCAGCGTAGACGCTT-3′, influenza M protein reverse 5′-CATCCTGTTGTATATGAGGCCCAT-3′; influenza M protein probe 5′-CTCAGTTATTCTGCTGGTGCACTTGCCA-3′ as described [14]. Alternatively, viral burden was measured by plaque assay using MDCK cells as described [15]. Relative expression of IFNλ2 and IFNλ3 was quantified by real-time PCR using the following primer sequences as described in [16]: IFNλ2 forward (59-AGGTGCAGTTCCCACCTCT-39) and reverse (59-TCAGTCATGTTCTCCCAGACC-39), IFNλ3 forward (59-TCCCAGCTGCAGACCTGT-39) and reverse (59-CAGGGGTCTCCTTGCTCTG-39), and were compared with HPRT as a reference gene.

### Statistical analysis

Data were analyzed using GraphPad Prism 7 (GraphPad, La Jolla, CA). All figures show combined data from multiple replicate studies as mean ± standard errors of the mean (SEM). The indicated n values are numbers of animals per independent experiment. Statistical significance was determined by Welch’s *t* test if comparing two groups of normally distributed data, one-way ANOVA if comparing more than two groups of normally distributed data, and by Kruskal-Wallis test for comparing more than two groups of non-normally distributed data. Significance is reported as * if *p* < 0.05. *p* values of between 0.05 and 0.1 are displayed numerically. For Fig 3, correlations were determined by linear regression, and exact *p* and R^2^ values are displayed.

## Results

IFNλ3^-/-^ mice were bred in-house and age- and sex-matched C57BL/6NJ WT controls were co-housed with IFNλ3^-/-^ mice for one week preceding influenza infection. Mice were infected with 25 PFU influenza A/PR/8/34 H1N1, six days later challenged with 5×10^7^ CFU USA300 MRSA, and harvested one day later. We saw no difference in bacterial burden or cellularity of the BAL as a measure of pulmonary inflammation (Fig 1A and 1B). Surprisingly, total IFNλ levels as measured by ELISA were no different between groups (Fig 1C). Importantly, murine IFNλ2 and IFNλ3 are almost identical in protein sequence [17], and there is no existing ELISA that is specific to a single murine IFNλ [18]. These data suggest that an increase in IFNλ2 compensated for the lack of IFNλ3 during influenza/bacterial super-infection. Moreover, influenza M protein expression was no different in the IFNλ3^-/-^ mice (Fig 1D), suggesting that a compensatory increase in IFNλ2 in the absence of IFNλ3 provided IFNλ3^-/-^ mice with the same level of anti-viral defense as WT mice.

Humans and mice have demonstrated sex differences in response to influenza, with females being more susceptible [19,20]. IFNλ3^-/-^ mice reproduced this difference in influenza/bacterial super-infection, with bacterial burden significantly lower in IFNλ3^-/-^ males as compared to females (Fig 2A). Similarly, WT males trended to have lower bacterial burden than WT females (Fig 2A), and there was a similar trend in BAL cellularity between sexes (Fig 2B). However, no differences existed between IFNλ3^-/-^ and WT mice in either bacterial burden or BAL cellularity, reinforcing that there is likely no difference in response to super-infection due to IFNλ3 deletion. Moreover, these mice exhibited no difference in BAL IFNλ, suggesting that females and males have equal ability to compensate for a lack of IFNλ3 with increased IFNλ2 expression (Fig 2C).

There was wide variation in the amount of total IFNλ in the bronchoalveolar lavage, regardless of sex or genotype. To investigate whether IFNλ production affected bacterial super-infection during influenza, we examined if there was a correlation between IFNλ production and super-infection outcomes. Strikingly, BAL IFNλ protein positively correlated with both BAL cellularity (Fig 3A) and bacterial burden (Fig 4C). Importantly, BAL IFNλ protein also positively correlated with influenza M protein expression (Fig 3B) regardless of genotype (Fig 3C), suggesting that a higher type III IFN response to influenza is associated with greater inflammation and bacterial burden during super-infection.

Surprisingly, while BAL IFNλ protein positively correlated with bacterial burden (Fig 4C), neither viral burden (Fig 4A) nor BAL cellularity (Fig 4B) were correlated with bacterial burden. It has long been thought that the severity of influenza infection affects susceptibility to secondary bacterial infection, but viral burden did not correlate with bacterial burden (Fig 4A). Cellular inflammation has been more strongly linked to bacterial susceptibility, as the highest susceptibility to super-infection temporally coincides with the peak of BAL cellularity in murine models, but cellular inflammation also did not correlate with bacterial burden (Fig 4B). These results suggest that IFNλ plays an important role in determining susceptibility to bacterial super-infection during influenza.

While IFNλ3^-/-^ mice and their WT controls were cohoused for one week preceding influenza infection (two weeks before sacrifice), we were suspicious that the observed sex differences might be due to the difference in cohousing techniques used between sexes. Cohousing was performed to normalize the gut microbiome between mice. As mice are coprophagic, placing mice of disparate genotypes in the same cage or switching bedding between cages serves to equalize their gut microbiota. While female mice can be placed in the same cage as other adult female mice, non-littermate male mice often fight, wounding each other and causing inflammation that alters experimental results. Thus, to cohouse males, mice were moved from their cage to one of the opposing genotype daily. By cohousing male mice in this manner, we observed a normalization of microbiome between the cages as evidenced by 16S sequencing of fecal samples (S1 Fig). Fully swapping bedding between cages should produce similar results to directly co-housing mice, however we did observe a difference in super-infection outcomes between the sexes, which suggested that these two methods may not generate equivalent effects on the murine gut microbiome.

**Fig 5:**
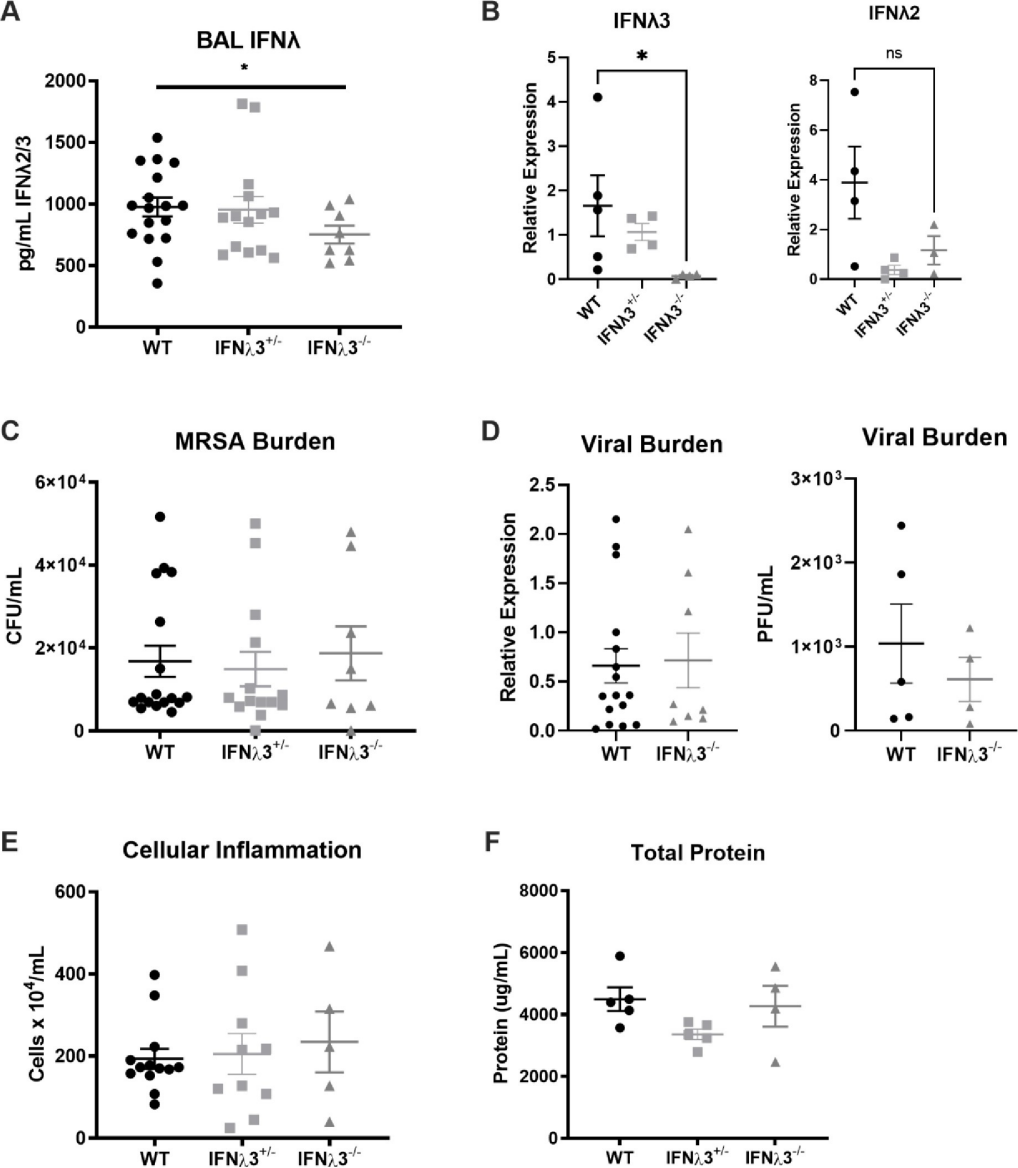
IFNλ3^-/-^ mice mount as effective of a response to influenza/bacterial super-infection as their WT littermates. IFNλ3^-/-^ females were bred with C57BL/6NJ males, and the entire F2 generation was subjected to influenza/bacterial super-infection (n = 3-13/group, two independent experiments). As previously, infected with 25 PFU influenza A/PR/8/34 H1N1, six days later challenged with 5×10^7^ CFU USA300 MRSA, and harvested one day following bacterial challenge. **(A)** BAL IFNλ was quantified by ELISA for IFNλ2/3. The difference between WT and IFNλ3^-/-^ mice was significant *p* < 0.05 cutoff when these two groups were analyzed by Welch’s *t* test, but were not significant when all three groups were analyzed by one-way ANOVA. **(B)** IFNλ2 and IFNλ3 were quantified by real-time PCR. **(C)** MRSA burden was measured by plating of lung homogenate and counting colony-forming units. **(D)** Viral burden was quantified by real-time qPCR for influenza M protein, and confirmed by plaque assay (n = 4-5/group, one experiment). **(E)** BAL cellularity was determined by counting total cells on a hemocytometer. **(F**) Lung inflammation was quantified by measuring total protein in the BAL by BCA assay.

To test this hypothesis, we bred C57BL/6NJ males purchased from Jackson Labs to IFNλ3^-/-^ females bred in a homozygous manner in-house, and used knockout and WT mice from the F2 generation to determine the effect of IFNλ3 gene composition on response to super-infection. Unlike the co-housed mice, IFNλ3^-/-^ mice had significantly reduced total IFNλ compared to their WT littermates (Fig 5A). Primers that differentiate IFNλ2 and IFNλ3 transcripts have recently been published [16]. We examined whether IFNλ transcript levels differed in IFNλ3^-/-^ mice. IFNλ3 transcript was undetectable in IFNλ3^-/-^ mice as expected. IFNλ2 transcript levels were not significantly reduced in IFNλ3^-/-^ mice (Fig 5B). This change in IFNλ expression did not result in altered response to bacterial super-infection (Fig 5C) possibly due to the small difference in total IFNλ between genotypes (975.379 to 752.081 pg/ml, Fig 5A). Moreover, viral burden was no different (Fig 5D), suggesting that the reduction in total IFNλ in IFNλ3^-/-^ mice as compared to their WT littermates is not enough to reproduce the differences in antiviral immunity seen in humans with IFNλ polymorphisms. There were no statistically significant differences in MRSA burden between the sexes, suggesting that the previously observed discrepancies were potentially due to contrasting co-housing methods (S2 Fig).

We then examined the impact of deletion of IFNλ3 on airway inflammation, lung pathology, and lung leak. We did not observe any difference in BAL cellularity or differential cell counts between genotypes (Figs 5E and S3). Interestingly, we did see increased perivascular inflammation, but not peribronchial or parenchymal, in IFNλ3^-/-^ mice compared with controls (S4 Fig). Finally there was no difference in lung leak of protein into the BAL regardless of genotype (Fig 5F). These data suggest that deletion of IFNλ3 may result in small changes in lung inflammation during super-infection.

## Discussion

Gene variants in interferon-stimulated genes (ISGs), the downstream effectors of interferons, can drastically impair the human response to influenza [21–24]. Type III interferons (IFNλ1–4) are polymorphically expressed in humans, with many individuals having an inactive IFNλ4 pseudogene [11]. Five single nucleotide polymorphisms (SNPs) have been identified in IFNλ3 with epidemiological implications for various viral infections including measles, herpes simplex virus, cytomegalovirus, Epstein-Barr virus, Andes virus, human T-lymphotropic virus type 1, and hepatitis B and C viruses. Importantly, expression of the IFNλ3 gene minor alleles (TG or GG) at rs8099917 was associated with increased seroconversion and increased Type 2 cytokine response to influenza H1N1 infection in an immunosuppressed population. Moreover, PBMCs from healthy minor-allele carriers stimulated with influenza H1N1 expressed less IFNλ3 than those from major-allele carriers [18]. Polymorphisms at rs8099917 in the IFNλ3 gene have also been associated with increased influenza-like illness symptoms during seasonal influenza H3N2 infection [25].

While IFNλ is a crucial part of the lung’s immunity to influenza, much less is known about its role in bacterial super-infection, which is a leading cause of influenza-associated mortality [5]. Only one paper thus far has observed an effect of IFNλ SNPs on bacterial infection, which suggests that polymorphisms in the IFNλ receptor gene are associated with differential response to lower urinary tract infection [26]. No epidemiological data exist that address the role of IFNλ SNPs in bacterial super-infection during influenza. Thus, we aimed to use IFNλ3^-/-^ mice to model a physiologically relevant reduction in IFNλ. However, IFNλ3^-/-^ mice did not display a marked reduction in total IFNλ2/3 protein when compared with co-housed C57BL/6NJ controls. Even when compared with WT littermate controls, the total amount of IFNλ2 and 3 in IFNλ3^-/-^ mice is not reduced sufficiently to model the reduction in antiviral immunity caused by human polymorphisms, as evidenced by lack of difference in their response to influenza. While IFNλ3^-/-^ do lack IFNλ3 transcript, they maintain expression of IFNλ2 as evidenced by real-time PCR and ELISA. Importantly, this resulted in no difference in either antiviral or antibacterial immunity during influenza infection. These data suggest that maintained IFNλ2 transcription in the absence of IFNλ3 is enough to protect mice from IFNλ2/3-dependent infections, and implies a functional redundancy between these two highly similar proteins.

Importantly, in both IFNλ3^-/-^ and WT mice, IFNλ levels in the bronchoalveolar lavage were positively correlated with influenza viral burden. As exogenous pegylated-IFNλ treatment has been shown to reduce influenza morbidity and mortality [1], these data suggest that a stronger induction of IFNλ is a response to higher viral burden, regardless of genotype. BAL IFNλ levels were also positively correlated with bacterial burden, suggesting that higher viral burden might drive higher bacterial burden. Interestingly, neither viral burden nor BAL cellularity directly correlated with bacterial burden. It has long been assumed that the severity of influenza infection affects susceptibility to bacterial super-infection. Moreover, while the timing of highest susceptibility to bacterial super-infection coincides with peak BAL cellularity, cellular inflammation was also not directly correlated with bacterial burden. However, viral burden positively correlated with cellular inflammation (S5 Fig), suggesting that higher viral burden may increase BAL cellularity. Together, these data suggest that IFNλ acts as a key mediator in these processes. We suggest a model in which higher viral burden drives increased cellular inflammation and IFNλ independent of each other, in which the IFNλ response to influenza is able to predict susceptibility to bacterial super-infection.

## Supporting information

S1 Fig Bedding swapping partially normalizes intestinal microbiome between cages of male mice. Bedding was swapped daily between cages of WT C57BL/6NJ mice (#1, 2, and 4) purchased from Taconic Biosciences and IFNλ3-/- mice (#5, 6, 8, here marked as "28BKO") bred at the University of Pittsburgh. A PCA plot was generated from 16S sequencing performed on fecal samples taken directly before co-housing, paired with samples from the same mice taken two weeks later at harvest.
(PDF)

S2 Fig Littermate WT and IFNλ3-/- mice do not exhibit sex differences in bacterial burden. IFNλ3-/- females were bred with C57BL/6NJ males, and the entire F2 generation was subjected to influenza/bacterial super-infection (n = 3-13/group, two independent experiments). As previously, infected with 25 PFU influenza A/PR/8/34 H1N1, six days later challenged with 5x10^7^ CFU USA300 MRSA, and harvested one day following bacterial challenge. MRSA burden was measured by plating of lung homogenate and counting colony-forming units.
(PDF)

S3 Fig Absence of IFNλ3 does not alter airspace-infiltrating cellular response to influenza/bacterial super-infection. Littermate WT, IFNλ3+/-, and IFNλ3-/- mice were infected with 25 PFU influenza A/PR/8/34 H1N1, six days later challenged with 5x10^7^ CFU USA300 MRSA, and harvested one day following bacterial challenge. At harvest, lungs were lavaged with 1 mL of sterile PBS, and the cells were processed via cytospin followed by modified Wright-Giemsa staining (Diff-Quik) for differential counting.
(PDF)

S4 Fig Absence of IFNλ3 may slightly increase histological inflammation in response to influenza/bacterial super-infection. Littermate WT, IFNλ3 +/-, and IFNλ3 -/- mice were infected with 25 PFU influenza A/PR/8/34 H1N1, six days later challenged with 5x10^7^ CFU USA300 MRSA, and harvested one day following bacterial challenge. At harvest, the left lobe of the lung was perfused with 10% formalin and later sectioned for histological determination of inflammation. Perivascular, peribronchial, and parenchymal inflammation were determined by blinded scoring of hematoxylin and eosin-stained sections by two independent investigators.
(PDF)

S5 Fig Influenza viral burden correlates with the number or inflammatory cells in the airspaces. IFNλ3-/- mice and C57BL/6NJ controls were co-housed for one week, infected with 25 PFU influenza A/PR/8/34 H1N1, six days later challenged with 5x10^7^ CFU USA300 MRSA, and harvested one day following bacterial challenge (n = 2-5/group, six independent experiments). Viral burden was quantified by real-time qPCR for influenza M protein, and BAL cellularity was determined by counting total cells on a hemocytometer.
(PDF)
