# Supplementary material for: Murine Type III interferons are functionally redundant and correlate with bacterial burden during influenza/bacterial super-infection
Source: PLoS One. 2021 Oct 7;16(10):e0255309. doi: 10.1371/journal.pone.0255309 (PMC8496871; doi:10.1371/journal.pone.0255309)
Supplement: S1 Fig — Bedding was swapped daily between cages of WT C57BL/6NJ mice (#1, 2, and 4) purchased from Taconic Biosciences and IFNλ3-/- mice (#5, 6, 8, here marked as "28BKO") bred at the University of Pittsburgh. A PCA plot was generated from 16S sequencing performed on fecal samples taken directly before co-housing, paired with samples from the same mice taken two weeks later at harvest. (PDF) [file pone.0255309.s001.pdf]

Supplemental Figure 1

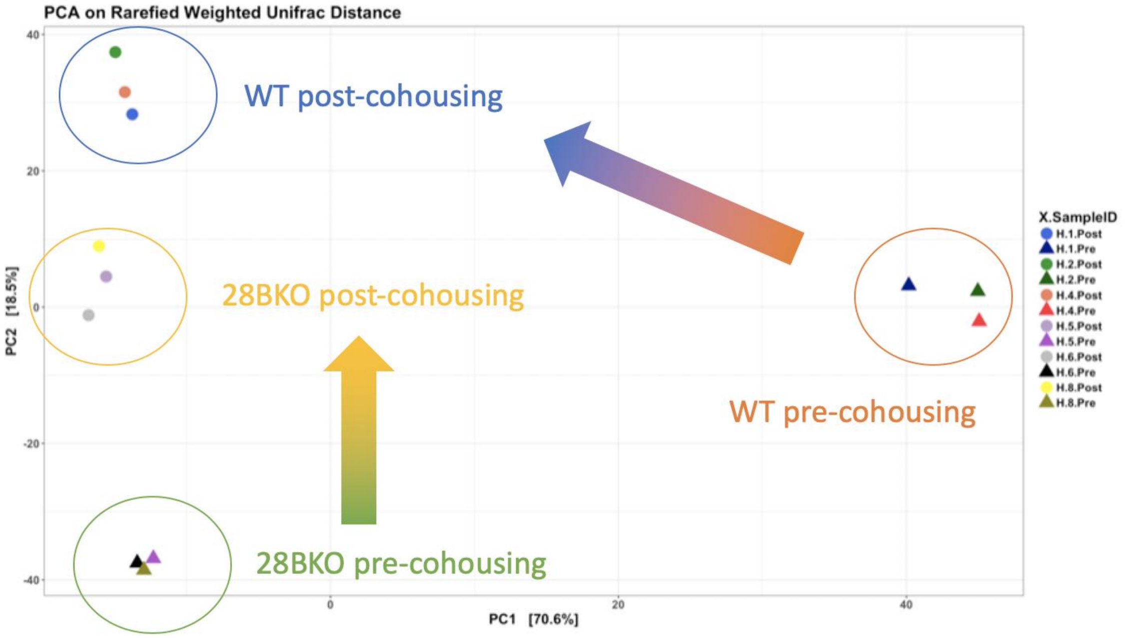

Figure S1. Bedding swapping partially normalizes intestinal microbiome between cages of male mice. Bedding was swapped daily between cages of WT C57BL/6NJ mice (#1, 2, and 4) purchased from Taconic Biosciences and IFNλ3-/- mice (#5, 6, 8, here marked as "28BKO") bred at the University of Pittsburgh. A PCA plot was generated from 16S sequencing performed on fecal samples taken directly before co-housing, paired with samples from the same mice taken two weeks later at harvest.
