# Supplementary material for: Murine Type III interferons are functionally redundant and correlate with bacterial burden during influenza/bacterial super-infection
Source: PLoS One. 2021 Oct 7;16(10):e0255309. doi: 10.1371/journal.pone.0255309 (PMC8496871; doi:10.1371/journal.pone.0255309)
Supplement: S2 Fig — IFNλ3-/- females were bred with C57BL/6NJ males, and the entire F2 generation was subjected to influenza/bacterial super-infection (n = 3-13/group, two independent experiments). As previously, infected with 25 PFU influenza A/PR/8/34 H1N1, six days later challenged with 5x10^7^ CFU USA300 MRSA, and harvested one day following bacterial challenge. MRSA burden was measured by plating of lung homogenate and counting colony-forming units. (PDF) [file pone.0255309.s002.pdf]

Supplemental Figure 2

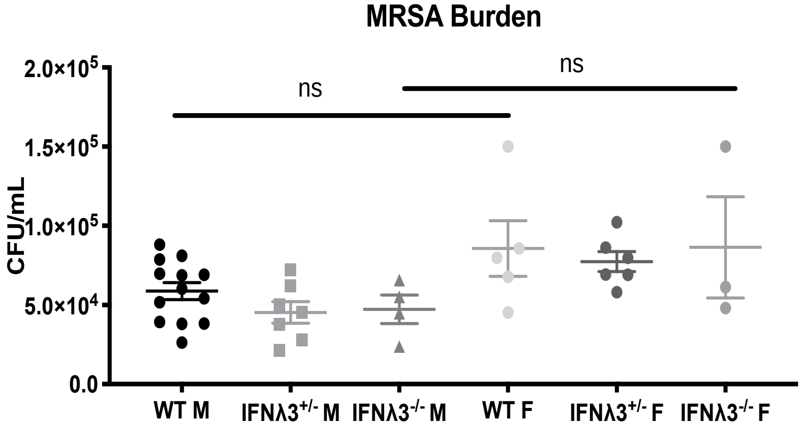

Figure S2. Littermate WT and IFNλ3-/- mice do not exhibit sex differences in bacterial burden. IFNλ3$^{-/-}$ females were bred with C57BL/6NJ males, and the entire F2 generation was subjected to influenza/bacterial super-infection (n = 3-13/group, two independent experiments). As previously, infected with 25 PFU influenza A/PR/8/34 H1N1, six days later challenged with 5×10^{7} CFU USA300 MRSA, and harvested one day following bacterial challenge. MRSA burden was measured by plating of lung homogenate and counting colony-forming units.
