# Supplementary material for: Murine Type III interferons are functionally redundant and correlate with bacterial burden during influenza/bacterial super-infection
Source: PLoS One. 2021 Oct 7;16(10):e0255309. doi: 10.1371/journal.pone.0255309 (PMC8496871; doi:10.1371/journal.pone.0255309)
Supplement: S3 Fig — Littermate WT, IFNλ3+/-, and IFNλ3-/- mice were infected with 25 PFU influenza A/PR/8/34 H1N1, six days later challenged with 5x10^7^ CFU USA300 MRSA, and harvested one day following bacterial challenge. At harvest, lungs were lavaged with 1 mL of sterile PBS, and the cells were processed via cytospin followed by modified Wright-Giemsa staining (Diff-Quik) for differential counting. (PDF) [file pone.0255309.s003.pdf]

Supplemental Figure 3

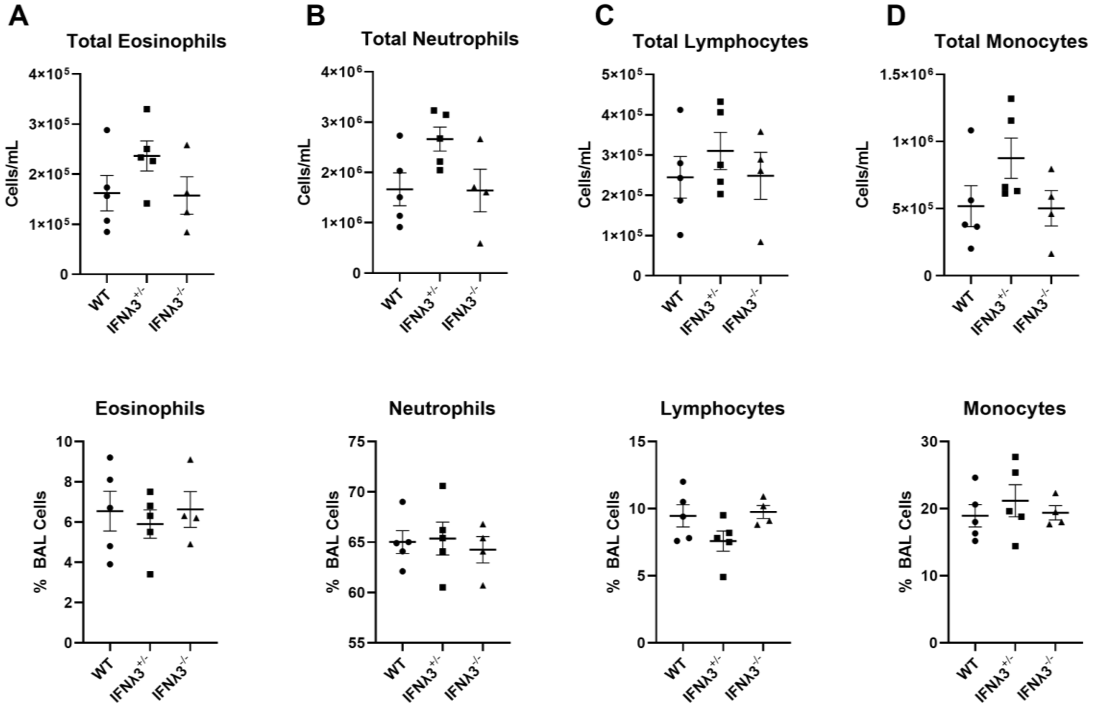

Figure S3. Absence of IFNλ3 does not alter airspace-infiltrating cellular response to influenza/bacterial super-infection. Littermate WT, IFNλ3+/-, and IFNλ3-/- mice were infected with 25 PFU influenza A/PR/8/34 H1N1, six days later challenged with $5x10^7$ CFU USA300 MRSA, and harvested one day following bacterial challenge. At harvest, lungs were lavaged with 1 mL of sterile PBS, and the cells were processed via cytospin followed by modified Wright-Giemsa staining (Diff-Quik) for differential counting.
