# Supplementary material for: Murine Type III interferons are functionally redundant and correlate with bacterial burden during influenza/bacterial super-infection
Source: PLoS One. 2021 Oct 7;16(10):e0255309. doi: 10.1371/journal.pone.0255309 (PMC8496871; doi:10.1371/journal.pone.0255309)
Supplement: S4 Fig — Littermate WT, IFNλ3 +/-, and IFNλ3 -/- mice were infected with 25 PFU influenza A/PR/8/34 H1N1, six days later challenged with 5x10^7^ CFU USA300 MRSA, and harvested one day following bacterial challenge. At harvest, the left lobe of the lung was perfused with 10% formalin and later sectioned for histological determination of inflammation. Perivascular, peribronchial, and parenchymal inflammation were determined by blinded scoring of hematoxylin and eosin-stained sections by two independent investigators. (PDF) [file pone.0255309.s004.pdf]

Supplemental Figure 4

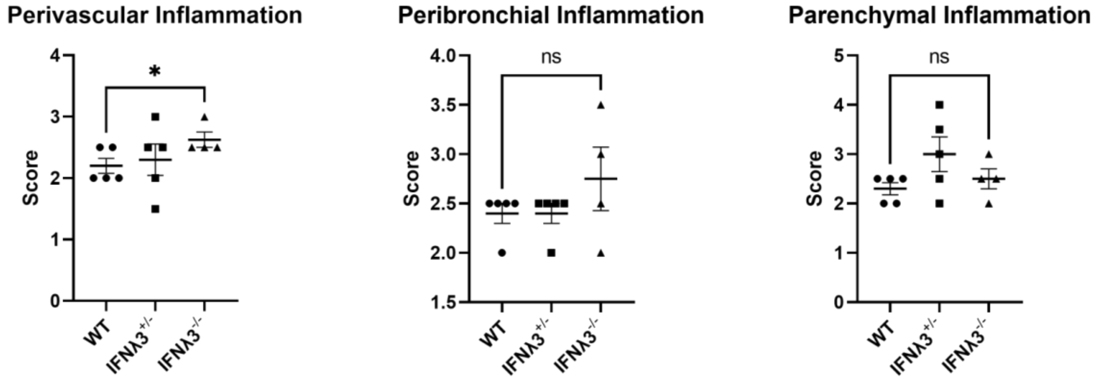

Figure S4. Absence of IFNλ3 may slightly increase histological inflammation in response to influenza/bacterial super-infection. Littermate WT, IFNλ3 +/-, and IFNλ3 -/- mice were infected with 25 PFU influenza A/PR/8/34 H1N1, six days later challenged with $5x10^7$ CFU USA300 MRSA, and harvested one day following bacterial challenge. At harvest, the left lobe of the lung was perfused with 10% formalin and later sectioned for histological determination of inflammation. Perivascular, peribronchial, and parenchymal inflammation were determined by blinded scoring of hematoxylin and eosin-stained sections by two independent investigators.
