# Supplementary material for: Murine Type III interferons are functionally redundant and correlate with bacterial burden during influenza/bacterial super-infection
Source: PLoS One. 2021 Oct 7;16(10):e0255309. doi: 10.1371/journal.pone.0255309 (PMC8496871; doi:10.1371/journal.pone.0255309)
Supplement: S5 Fig — IFNλ3-/- mice and C57BL/6NJ controls were co-housed for one week, infected with 25 PFU influenza A/PR/8/34 H1N1, six days later challenged with 5x10^7^ CFU USA300 MRSA, and harvested one day following bacterial challenge (n = 2-5/group, six independent experiments). Viral burden was quantified by real-time qPCR for influenza M protein, and BAL cellularity was determined by counting total cells on a hemocytometer. (PDF) [file pone.0255309.s005.pdf]

Supplemental Figure 5

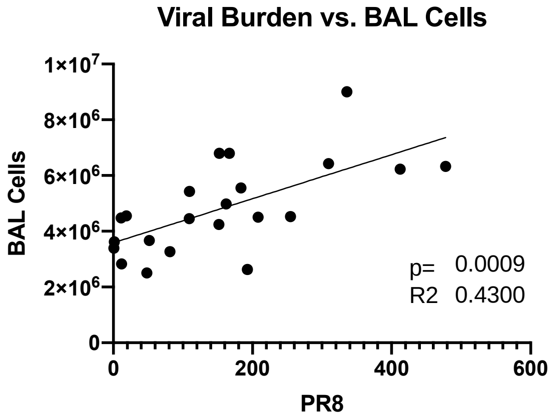

Figure S5. Influenza viral burden correlates with the number or inflammatory cells in the airspaces. IFNλ3$^{-/-}$ mice and C57BL/6NJ controls were co-housed for one week, infected with 25 PFU influenza A/PR/8/34 H1N1, six days later challenged with 5×10^7 CFU USA300 MRSA, and harvested one day following bacterial challenge (n = 2-5/group, six independent experiments). Viral burden was quantified by real-time qPCR for influenza M protein, and BAL cellularity was determined by counting total cells on a hemocytometer.
